# A randomized, controlled, multicenter phase III clinical trial of Huo Xiang Zheng Qi oral liquid for the prevention and control of nausea and vomiting caused by multiday cisplatin-based regimen

**DOI:** 10.1097/MD.0000000000046778

**Published:** 2025-12-26

**Authors:** Liping Tong, Songze Wu, Zixuan Ye, Ying Wang, Juan Chen, Ting Li, Xianguo Liu, Na Li, Taifang Peng, Yangang Zhou, Liqin Xia, Zengjin Hu, Zhiying Yue, Jie Xian, Jun He, Lang He, Yu Sun, Jiang Zhu

**Affiliations:** a Division of Thoracic Tumor Multimodality Treatment and Department of Medical Oncology, Cancer Center, West China Hospital, Sichuan University/Department of medical oncology, Shangjin Nanfu Hospital, Chengdu, Sichuan, China; b Department of Respiratory and Critical Care Medicine, State Key Laboratory of Respiratory Health and Multimorbidity, West China Hospital, Sichuan University, Chengdu, Sichuan, China; c Division of Thoracic Tumor Multimodality Treatment and Department of Medical Oncology, Cancer Center, West China Hospital, Sichuan University, Chengdu, Sichuan, China; d Department of Oncology, Cancer Prevention and Treatment Institute of Chengdu, Chengdu Fifth People’s Hospital (The Second Clinical Medical College, Affiliated Fifth People’s Hospital of Chengdu University of Traditional Chinese Medicine), Chengdu, Sichuan, China; e Cancer Center of 903 Hospital, City of Jiangyou, Sichuan, China; f Department of oncology, the second people’s hospital of Yibin, Yibin, Sichuan, China; g Department of Oncology, The Affiliated Chengdu 363 Hospital of Southwest Medical University, Chengdu, Sichuan, China; h Department of Oncology, Suining Central Hospital, Suining, Sichuan, China; i Chongqing University Qianjiang Hospital/ Qianjiang Central Hospital of Chongqing, Chongqing, China; j Oncology Department, The First Afffliated Hospital of Chengdu Medical College, Chengdu, Sichuan, China; k West China-Guang’An Hospital, Sichuan University, Guang’an, Sichuan, China; l Oncology Department, Qianjiang National Hospital, Qianjiang, Chongqing, China; m Department of Biotherapy Research, Cancer Center, West China Hospital, Sichuan University, Chengdu, Sichuan, China; n Radiotherapy Physics and Technology Center, Cancer Center, West China Hospital, Sichuan University, Chengdu, Sichuan, China

**Keywords:** Chemotherapy-induced nausea and vomiting (CINV), highly emetogenic chemotherapy (HEC), Huo Xiang Zheng Qi Oral Liquid (HXZQ), multiday cisplatin

## Abstract

**Background:**

Standardized chemotherapy-induced nausea and vomiting (CINV) prevention in HEC is critical, yet NK1RAs remain inaccessible for some patients due to cost and availability. Our prior study demonstrated HXZQ + 5HT3RAs + dexamethasone’s superior efficacy; this phase III trial aimed to validate this regimen.

**Method:**

This multicenter, double-blind, randomized phase III trial (10 hospitals, Southwest China; March 2023–August 2024) assigned patients to group HX (HXZQ + 5HT3RA + dexamethasone) or group C (control). Primary endpoints: mean No CINV Days (NCDs) during the full cycle and complete control (CC) rate beyond the risk period. Secondary endpoints: safety, CC rate during the risk period, mean no nausea days and life function.

**Results:**

A total of 166 patients were enrolled and 139 patients completed the study, 73 in group HX and 66 in group C. The mean NCDs was significantly better in group HX (17.92 ± 4.06) than that in group C (15.26 ± 5.91, *P* = .002). Group HX showed better NCDs in acute, delayed and the period beyond the risk phases than group C, with a higher CC rate of CINV beyond the risk phase (80.8% vs 60.6%, *P* = .009). The mean no nausea days in group HX was significantly better than that in group C (18.26 vs 15.45, *P* = .001). Group HX also showed a trend to better functional living index-emesis score, but only achieved the significance during the period beyond the risk phase.

**Conclusion:**

HXZQ in combination with a 5-HT3 receptor antagonist and dexamethasone is safe and feasible for preventing CINV due to 3-day cisplatin-containing HEC throughout the whole cycle.

## 1. Introduction

Despite advances in cancer treatment, chemotherapy remains a cornerstone for many cancers.^[1]^ However, chemotherapy-induced nausea and vomiting (CINV) persists as a common and distressing adverse reaction.^[2]^ Mechanisms include chemotherapy-generated free radicals triggering serotonin (via 5-HT3) and substance P (via NK-1) pathways, driving peripheral/central vomiting. Non-chemotherapy factors (e.g., gastric obstruction, brain metastases, anxiety) also contribute.^[3]^ A large number of Phase III clinical studies have demonstrated that the prophylactic administration of dexamethasone, 5-HT3 receptor antagonists, and NK-1 receptor antagonists, either as monotherapy or in combination with olanzapine, results in a 70% to 80% complete control (CC) rate for CINV.^[4,5]^ This regimen is currently recommended by guidelines for the prevention and treatment of HEC-induced CINV.^[5,6]^ However, real-world adherence to antiemetic guidelines remains suboptimal, particularly due to the low utilization of NK-1RAs.^[7,8]^ The results of the preliminary survey indicate that a significant proportion of patients in China who did not use NK1RAS cited accessibility issues and economic constraints. Furthermore, the management of CINV that persists over several days is a more intricate matter. The potential for superimposed acute CINV and delayed CINV complicates the prevention and control of CINV.^[9,10]^ The study group’s preliminary findings suggest that the occurrence of long-delayed CINV outside of the risk period is also a possibility following multiday HEC chemotherapy, with the potential to further impact patients’ quality of life.^[9,10]^ Therefore, in response to the above, it is necessary to explore other potentially effective CINV prophylaxis options for patients treated with multiday HEC chemotherapy and unavailable NK1RAs.

Over the past century, cancer therapy has evolved from surgery and radiotherapy to systemic treatments such as cytotoxic chemotherapy, targeted therapy, and immunotherapy, greatly improving survival but also introducing new challenges – especially treatment-related toxicities like nausea, fatigue, and gastrointestinal dysfunction, which continue to compromise patients’ quality of life.^[11]^ This has led to the development of modern supportive oncology, emphasizing symptom prevention and holistic care throughout treatment. Within this framework, Traditional Chinese Medicine (TCM) has gained growing recognition as a complementary approach in supportive cancer management. As summarized by Liu et al., TCM is widely applied in East Asian countries and has shown potential to alleviate symptoms, reduce chemotherapy-related adverse effects, and improve psychological well-being and treatment tolerance.^[12]^

According to TCM theory, nausea and vomiting are regarded as manifestations of “spleen–stomach disharmony” and “reversed qi flow” (pi-wei bu he, qi ni). Classical texts such as the Huangdi Neijing and Treatise on Cold Damage Disorders describe vomiting as a result of disrupted qi dynamics in the stomach and spleen, often involving a mixture of cold and heat. In TCM, the spleen and stomach are viewed as the foundation of digestion and energy transformation. When their ascending and descending functions are disturbed, internal retention of food or phlegm, external evils invading the stomach, or liver-qi stagnation may trigger rebellious stomach qi that ascends abnormally, leading to nausea and vomiting. In the modern era, chemotherapy is considered a potent form of yao du (“medicinal toxin”) that damages the spleen and stomach, disturbing qi movement and creating a pattern of combined cold and heat.^[13]^ Therefore, CINV are understood in TCM as a manifestation of toxin-induced spleen–stomach dysfunction. From a modern pharmacologic perspective, these symptoms are mediated primarily by serotonin (5-HT₃) and substance P/neurokinin-1 (NK₁) receptor pathways, which regulate gastrointestinal motility and the central vomiting reflex. This conceptual convergence – where restoring qi harmony in TCM parallels modulation of neuro-gastrointestinal signaling in Western medicine – provides a theoretical rationale for integrating TCM formulations into antiemetic strategies.^[3]^

In China, Huo Xiang Zheng Qi Oral Liquid is a widely used pharmaceutical preparation for the treatment of vomiting and diarrhea.^[14]^ Preliminary studies conducted by the research group indicate that the combination of dexamethasone and 5HT3RAs with Huo Xiang Zheng Qi Oral Liquid can effectively prevent CINV in patients undergoing 3-day cisplatin-based chemotherapy regimens.^[15]^ To further validate the role of Huo Xiang Zheng Qi Oral Liquid in the overall management of CINV, we conducted this prospective, multicenter, randomized placebo-controlled phase III clinical study.

## 2. Methods

### 2.1. Research design

This is a multicentre, prospective, double-blind, phase III randomized controlled trial. The study protocol and informed consent form followed ethical requirements and were approved by the Ethics Committee of West China Hospital of Sichuan University. The study had been registered in the China Clinical Trial Registry (registration number: ChiCTR2300069212).

### 2.2. Patients

The study was planned to be carried out in ten cancer centers in China: the Fifth People’s Hospital of Chengdu, the First Affiliated Hospital of Chengdu Medical College, the People’s Hospital of Guangan City, Jiangyou 903 Hospital, the Affiliated 363 Hospital of Southwest Medical University (Chengdu), the West China Shangjin Hospital of Sichuan University, the Central Hospital of Suining City, the Second People’s Hospital of Yibin City, the Qianjiang National Hospital of Chongqing, and the Central Hospital of Qianjiang, Chongqing. All enrolled patients provided written informed consent.

The following criteria were met by patients included in this study: Patients with a pathologically confirmed diagnosis of malignancy. Aged 18 to 70 years. Chemotherapy naive, or with prior adjuvant chemotherapy completed more than 6 months before enrollment. Chemotherapy regimen included 3-day cisplatin, alone or in combination with a single-agent regimen (cetuximab, trastuzumab, rituximab, bevacizumab, ICIs immunotherapy agents were allowed. TKIs are not permitted). A history of chronic gastrointestinal disease or chronic nausea and vomiting is not recorded. The patient can take HXZQ oral solution. The results of auxiliary examinations and important organ indexes meet the requirements for chemotherapy. The use of NK1RAs for prophylactic antiemetic purposes was contraindicated for various reasons.

The exclusion criteria encompassed the following: Patients experiencing uncontrolled cancer pain requiring morphine dose titration or adjustment of opioid analgesics were excluded from the study. Patients who had undergone long-term corticosteroid therapy were excluded from participation. Nausea and vomiting from any cause occurring within the previous 3 days. Patients with combined peptic ulcers, gastrointestinal bleeding, or other chronic diseases that result in nausea and vomiting. Patients with uncontrolled brain metastases or symptoms of intracranial hypertension. Patients unable to tolerate HXZQ Oral Liquid. Patients who, in the investigator’s judgment, were unable to complete the study as planned.

### 2.3. Grouping and blind methods

Eligible patients were randomized (1:1) to group HX or group C via computer-generated study identification numbers (IDs) (n = 166). Group allocation was concealed from both participants and researchers. Participants received sequential IDs and corresponding treatment assignments. The Cancer Psychology and Health Management Committee of the Sichuan Cancer Society oversaw the process, and the study was double-blind for both subjects and investigators.

### 2.4. Interventions

Patients in group HX were administered dexamethasone 6 mg QD, D1-4 + 5-HT3RAS + HXZQ Oral Liquid 10ml, TID, D1-21. Patients in group C were administered dexamethasone 6 mg QD, D1-4 + 5-HT3RAs + placebo 10ml, TID, D1-21.

HXZQ Oral Liquid consists of extracts of 10 herbs: Atractylodes macrocephala, Orange peel, Magnolia officinalis (ginger), Angelica dahurica, Poria Tuckahoe, raw pinellia, licorice extract, patchouli oil, Perilla leaf oil, and the excipients included polysorbate-80 and cinnamon oil. HXZQ Oral Liquid is approved by the State Food and Drug Administration and produced by Taiji Group Chongqing Fuling Pharmaceutical Co. in accordance with the 2020 edition of the Pharmacopoeia of the People’s Republic of China, the monographs. Placebo comprised ginger powder/liquid flavoring, stevioside, sucrose octaacetate, caramel color, and piperine, which matched HXZQ in appearance/taste.

Patients maintained daily diaries (Days 1–21 of chemotherapy) documenting nausea severity, vomiting frequency, and rescue antiemetic use. Diaries were distributed pre-chemotherapy, completed daily until Day 22, and submitted before the next cycle. The FLIE scale was self-administered (with investigator guidance) on Days 8 and 22 to assess CINV’s impact on daily function.

### 2.5. Research tools

A data collection form was employed to obtain baseline demographic characteristics from the HIS system, such as age, sex, cancer type, stage, ECOG score, history of smoking, history of brain metastases, history of alcoholism, history of cervical spondylosis, initial treatment or recurrence, gastrointestinal complications, history of oncologic surgery, history of cerebrovascular disease, and treatment regimen.

Patient diaries, maintained by the researchers, documented symptoms of nausea and vomiting.^[15]^ The patient diary was based on the mascc antiemetic tool.^[16]^ The mascc antiemetic tool required recording of daily vomiting episodes, nausea severity (0–10 visual analog scale), and use of rescue therapy. Clinicians provided diaries before chemotherapy, instructed patients to record from Day 1 to 21, and collected them before the next cycle.

Life function was assessed via the Functional Living Index-Emesis (FLIE),^[17]^ comprising nausea/vomiting domains (9 items each). Items used a 7-point visual analogue scale (1 = a lot, 7 = not at all). Domain scores summed items within each domain. The total FLIE score combined nausea/vomiting domain scores, with higher values indicating better quality of life and daily function. Scores >6 on a 7-point scale were deemed to have no effect on daily life. Patients completed the documentation questionnaire on days 7 and 21, corresponding to the effect of CINV on life functioning in subjects at risk period and the beyond-risk period respectively.

## 3. Statistical analysis

### 3.1. Sample size

In the preliminary study of Huo Xiang Zheng Qi Oral Liquid, it was established that the mean No CINV (NCDs) in patients undergoing highly emetogenic chemotherapy with the standard 2-drug antiemetic regimen was 12.13 ± 7.63. It was hypothesized that the combination of Huo Xiang Zheng Qi Oral Liquid would augment the mean NCDs by 4 days, with a significance level of 0.05 and a power of 0.90. In summary, the sample size of the study group (group HX) was calculated to be 83 cases, and 83 cases in the control group (group C), considering a 20% shedding rate, resulting in a total sample size of 166 cases.

### 3.2. Research endpoints

The primary endpoints of this research were the mean NCDs during the full cycle and the CC rate of CINV beyond the risk period. NCDs were defined as a day in which the patient did not experience any level of vomiting and nausea and had no relieving treatment. The day was then deemed as an NCD, and the cumulative sum of all of the above NCDs in a given period was deemed as the NCDs in this period.^[9,10]^ The CC rate of CINV beyond the risk period was defined as follows: The patient did not experience any level of vomiting in a given period, did not experience significant nausea, and did not experience rescue treatment. Secondary endpoints encompassed safety, the CC rate of CINV during the risk period, the mean No Nausea Days (NNDs), and life function.

### 3.3. Software

Statistical analyses were performed using SPSS 26.0 (IBM Corporation, Chicago). Continuous variables were compared via t-tests or Mann–Whitney *U* tests, and categorical variables via chi-square tests. The significance level was set at α = 0.05, and *P* < .05 was considered statistically significant.

## 4. Results

### 4.1. Patients’ characteristics

**Figure 1.**
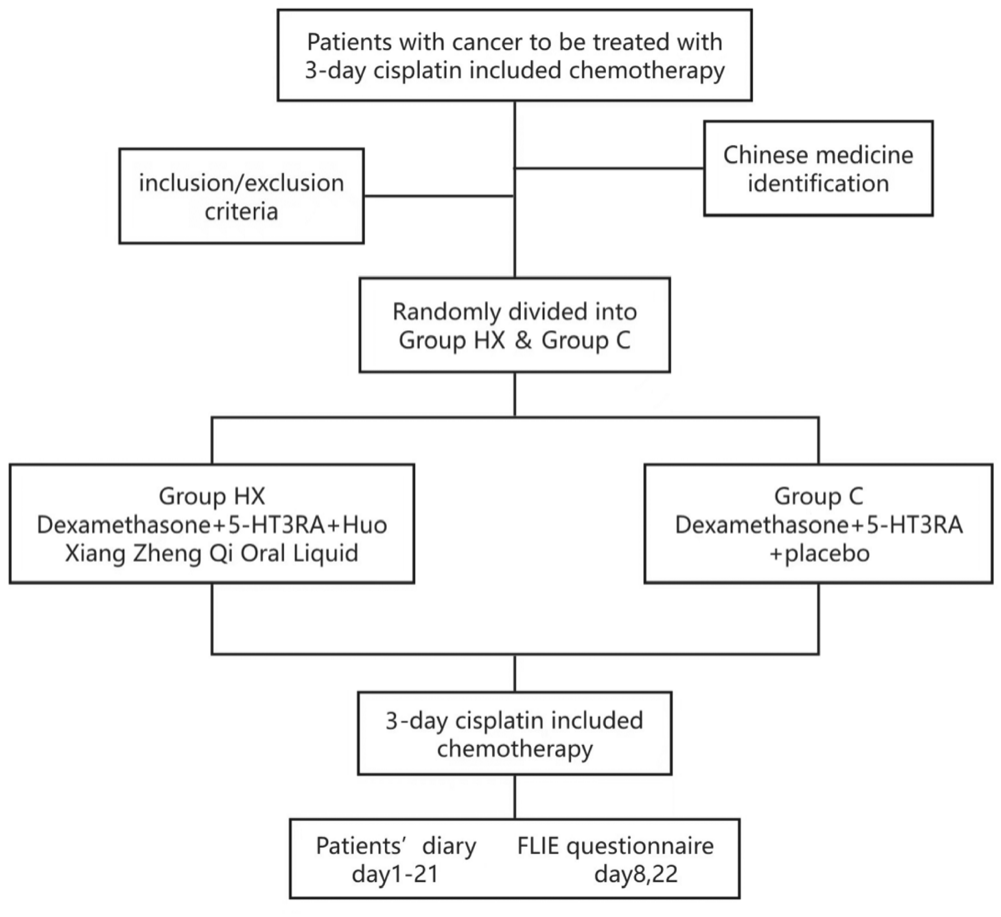
The flow chart.

From March 14, 2023 to August 2, 2024, patients were screened at ten oncology centers in Western China. A total of 166 patients who met the inclusion criteria were enrolled in the study. Participants were randomized in a 1:1 ratio to group HX (n = 83) or group C (n = 83). Ultimately, 139 patients completed the study. Twenty-seven patients (16.3%) discontinued participation, primarily due to intolerance to the odor or taste of HXZQ/placebo (n = 20, 12.0%), consent withdrawal, or incomplete data. In total, 139 patients (83.7%) completed the study (HX: 73; C: 66) and were included in the analysis (Fig. 1). Baseline characteristics are summarized in Table 1.

**Table 1:** Baseline characteristics.

Characteristic	Group HX(N = 73)	Group C(N = 66)	*P*-value
Age (year), mean (SD)	58.25 (9.51)	58.39 (7.78)	.921
Sex, n(%)			
Male	55 (75.30)	40 (60.60)	.062
Female	18 (24.70)	26 (39.40)	
Type of cancer, n(%)			
Lung cancer	40 (54.80)	37 (56.10)	.175
Esophageal cancer	9 (12.30)	7 (10.60)	
Liver cancer	0 (0.00)	1 (1.50)	
Nasopharyngeal cancer	6 (8.20)	7 (10.60)	
Gallbladder cancer	0 (0.00)	1 (1.50)	
Ovarian cancer	1 (1.40)	2 (3.00)	
Cervical cancer	6 (8.20)	9 (13.60)	
Others	11 (15.10)	2 (3.00)	
Stage, n(%)			
I	6 (8.20)	6 (9.10)	.931
II	5 (6.80)	6 (9.10)	
III	24 (32.90)	19 (28.80)	
IV	38 (52.10)	35 (53.00)	
ECOG score			
0	39 (53.40)	35 (53.00)	1.000
1	28 (38.40)	26 (39.40)	
2	5 (6.80)	5 (7.60)	
3	1 (1.40)	0 (0.00)	
History of smoking status, n(%)			
No	42 (57.50)	39 (59.10)	.865
Yes	31 (42.50)	27 (40.90)	
Brain metastasis status, n(%)			
No	67 (91.80)	63 (95.50)	.498
Yes	6 (8.20)	3 (4.50)	
History of alcoholism, n(%)			
No	68 (93.20)	61 (92.40)	1.000
Yes	5 (6.80)	5 (7.60)	
Previous adjuvant chemotherapy, n(%)			
No	59 (80.80)	49 (74.20)	.416
Yes	14 (19.20)	17 (25.80)	
History of cerebrovascular disease, n(%)			
No	71 (97.30)	65 (98.50)	1.000
Yes	2 (2.70)	1 (1.50)	
Chemotherapy regimen, n(%)			
Paclitaxel + Cisplatinum	45 (61.60)	31 (47.00)	.129
Gemcitabine + Cisplatinum	2 (2.70)	4 (6.10)	
Irinotecan + Cisplatinum	1 (1.50)	1 (1.40)	
Pemetrexed + Cisplatinum	19 (26.00)	14 (21.20)	
Docetaxel + Cisplatinum	1 (1.40)	3 (4.50)	
Etoposide + Cisplatinum	4 (5.50)	12 (18.20)	
Capecitabine + Cisplatinum	1 (1.40)	1 (1.50)	

ECOG = Eastern Cooperative Oncology Group.

### 4.2. Safety

In the present study, no fatalities related to the study drug were observed, and no treatment-related adverse events were recorded. However, some subjects indicated that the odor or taste of the HXZQ liquid and the placebo could cause discomfort (11, 13.3%) cases in the group HX and 9 (10.8%) cases in the group C, *P* = .83). The most frequent adverse events in both groups were fatigue, constipation, neutropenia and anemia. The occurrence of adverse reactions is shown in Table 2.

**Table 2:** Adverse events.

Events	Grade 1		Grade2		Grade 3		*P*-value	TRAE (Y/N)*
	Group HX (N = 73)	Group C (N = 66)	Group HX (N = 73)	Group C (N = 66)	Group HX (N = 73)	Group C (N = 66)		
Neutropenia	23 (31.5)	18 (27.3)	10 (13.7)	13 (19.7)	1 (1.4)	3 (4.5)	0.514	N
Thrombocytopenia	12 (16.4)	7 (10.6)	4 (5.5)	3 (4.5)	0 (0.0)	2 (3.0)	0.416	N
Anemia	17 (23.3)	20 (30.3)	9 (12.3)	9 (13.6)	2 (2.7)	1 (1.5)	0.731	N
Liver injury	7 (9.6)	6 (9.1)	1 (1.4)	0 (0.0)	0 (0.0)	0 (0.0)	1.000	N
Renal injury	0 (0.0)	1 (1.5)	2 (2.7)	0 (0.0)	0 (0.0)	0 (0.0)	0.356	N
Palpitation	2 (2.7)	0 (0.0)	0 (0.0)	0 (0.0)	–	–	0.498	N
Constipation	21 (28.8)	21 (31.8)	5 (6.8)	3 (4.5)	0 (0.0)	0 (0.0)	0.845	NA**
Diarrhea	3 (4.1)	0 (0.0)	1 (1.4)	1 (1.5)	0 (0.0)	1 (1.5)	0.210	NA
Stomachache	2 (2.7)	3 (4.5)	1 (1.4)	0 (0.0)	0 (0.0)	0 (0.0)	0.827	NA
Fatigue	24 (32.9)	19 (28.8)	11 (15.1)	10 (15.2)	0 (0.0)	0 (0.0)	0.894	N
Hiccup	8 (11.0)	2 (3.0)	0 (0.0)	0 (0.0)	0 (0.0)	0 (0.0)	0.101	NA
Cough	13 (17.8)	6 (9.1)	1 (1.4)	2 (3.0)	0 (0.0)	1 (1.5)	0.302	N
Fever	0 (0.0)	2 (3.0)	1 (1.4)	0 (0.0)	0 (0.0)	0 (0.0)	0.224	N
Parageusia	4 (5.5)	5 (7.6)	0 (0.0)	0 (0.0)	0 (0.0)	0 (0.0)	0.736	Y
Hair loss	11 (15.1)	13 (19.7)	3 (4.1)	7 (10.6)	–	–	0.214	N
Skin rash	2 (2.7)	0 (0.0)	0 (0.0)	1 (1.5)	0 (0.0)	0 (0.0)	0.356	NA

* TRAE: treatment-related adverse event.

** NA: not assessable.

### 4.3. The mean no CINV days

**Figure 2.**
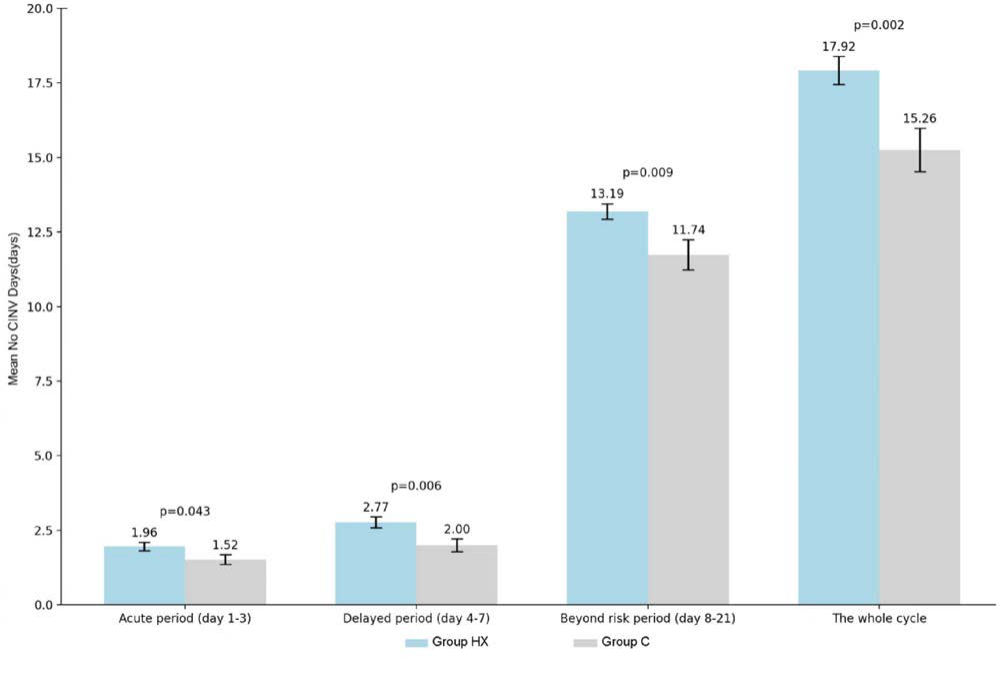
The average no CINV days in the acute, delayed and beyond the risk phase. CINV = chemotherapy-induced nausea and vomiting.

During the entire chemotherapy cycle, the mean number of NCDs in the group HX was 17.92 ± 4.06, which was significantly better than that in the group C (15.26 ± 5.91, *P* = .002). The mean NCDs in the acute, delayed and beyond-risk phases in group HX were 1.96 ± 1.23, 2.77 ± 1.58 and 13.19 ± 2.23 respectively, which were significantly better than those of the group C (1.52 ± 1.33, 2.00 ± 1.69, and 11.74 ± 4.06; *P* = .043, 0.006 and 0.009) (Fig. 2).

### 4.4. The complete control rate of CINV beyond the risk period

**Figure 3.**
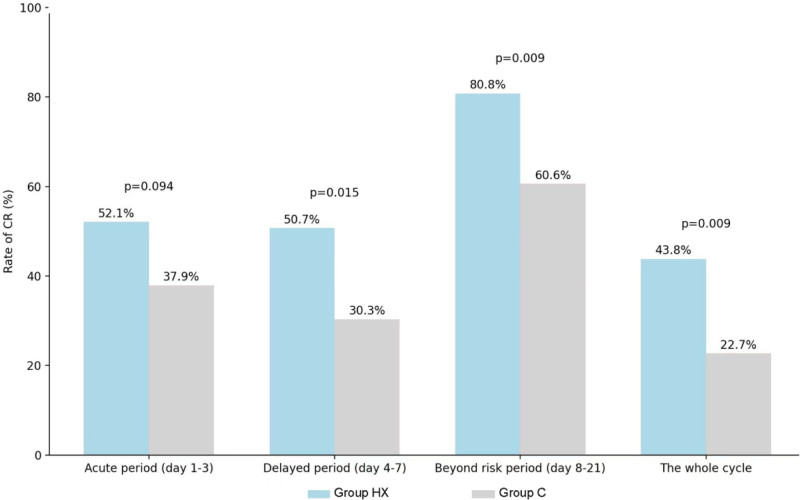
CR rate of CINV in the acute, delayed and beyond the risk phase. CINV = chemotherapy-induced nausea and vomiting, CR = complete remission.

The CC rate of CINV beyond the risk period in the group HX was 80.8%, which was significantly higher than that in group C (60.6%, *P* = .009). Furthermore, the CC rate of CINV in the full cycle and delayed phase in the group HX were 43.8% and 50.7%, which were significantly higher than those in the group C (22.7%, *P* = .009; 30.3%, *P* = .015). The CC rate of CINV in the acute phase in the group HX (52.1%) was also higher than that in the group C (37.9%), but this difference was not statistically significant (Fig. 3).

### 4.5. The average no nausea days

**Figure 4.**
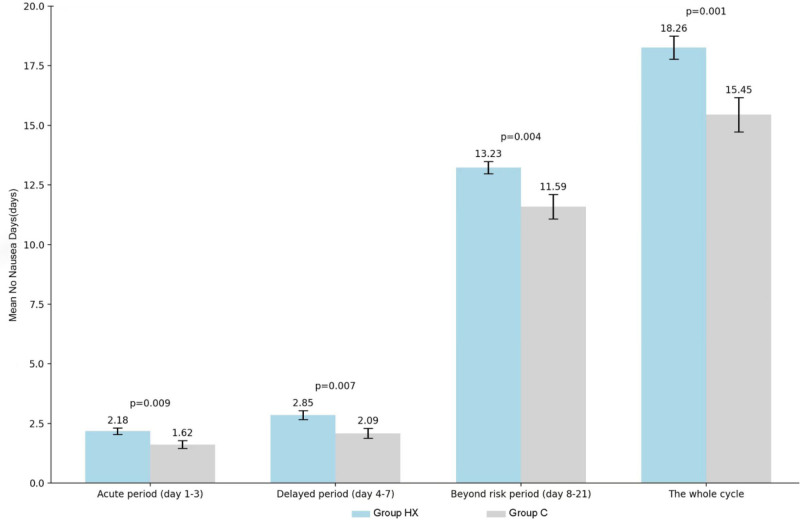
The average no nausea days in the acute, delayded and beyond the risk phase.

The mean NNDs during the full cycle in group HX was 18.26, which was significantly higher than that in group C (15.45 ± 5.87, *P* = .001). Conversely, the mean NNDs beyond the risk period in the group HX was 13.23 ± 2.23, which was also significantly better than that in the group C (11.59 ± 4.16, *P* = .004). During the risk period in group HX, the mean NNDs in the acute and delayed phases were 2.18 ± 1.16 and 2.85 ± 1.57, respectively, which were significantly higher than those in the group C (1.62 ± 1.31 and 2.09 ± 1.72; *P* = .009 and 0.007) (Fig. 4).

### 4.6. Life function

**Figure 5.**
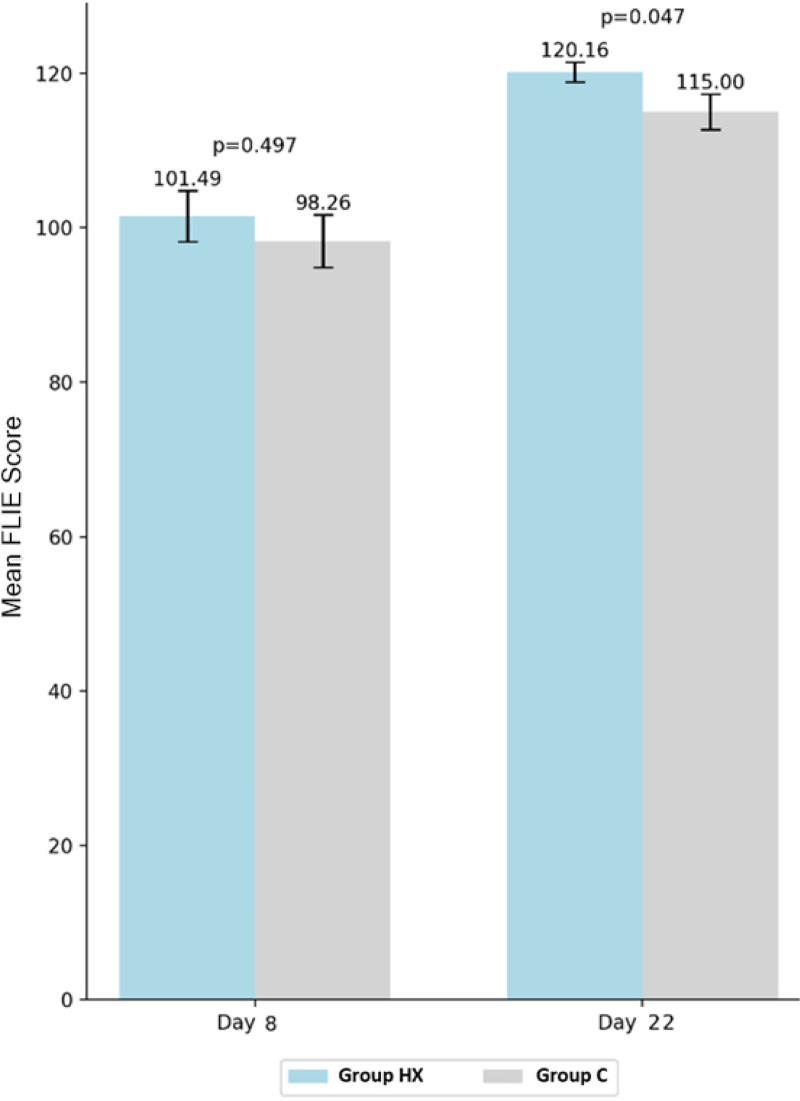
Functional Living Index-Emesis (FLIE) questionnarie score at day 8 and day 22.

According to the FLIE scale score on day 7 of chemotherapy, the mean score of patients in the group HX was 101.49 ± 28.36, which was higher than that of patients in the group C (98.26 ± 27.52), but the difference did not reach statistical significance (*P* = .497). On day 21, the mean score in the group HX was 120.16 ± 11.12, which was significantly higher than that in the group C (115.00 ± 18.66, *P* = .047) (Fig. 5).

## 5. Discussion

Nausea and vomiting, commonly experienced by cancer patients undergoing chemotherapy, represent a significant reduction in quality of life during antineoplastic therapy. The development of effective prevention strategies for healthcare providers and related organizations has been facilitated by ASCO, NCCN, and MASCC/ESMO CINV practice guidelines. Nevertheless, the real-world clinical picture is less than satisfactory, and CINV continues to be a significant problem for cancer patients undergoing antineoplastic therapy.^[8,18,19]^

This study further investigates the feasibility and safety of an HXZQ-containing regimen for the prevention and treatment of CINV. This investigation is based on a phase IIB clinical trial of HXZQ oral solution^[15]^ in combination with 5-HT3 RA and dexamethasone. The study revealed that the combination of HXZQ oral solution with 5-HT3 RA and dexamethasone led to a 15% to 20% increase in the rate of CC of CINV during the risk period, as well as a 20% increase in the rate of CC of CINV beyond the risk period. The mean No CINV during the entire cycle was found to be 17.92 ± 4.06 in the group HX, which was significantly higher than the 15.26 ± 5.91 observed in the group C. The number of days in the acute phase and delayed phase, as well as the mean No CINV beyond the risk period, were observed to be 1.96 ± 1.23 vs 1.52 ± 1.33, 2.77 ± 1.58 vs 2.00 ± 1.69, and 13.19 ± 2.23 vs 11.74 ± 4.06. The group HX demonstrated a significantly higher FLIE total score than the group C, indicating an enhancement in quality of life. Furthermore, the group HX exhibited a lower incidence of CINV for 6 days beyond the acute, delayed, and risk phases, compared with group C. These results suggest that HXZQ oral solution combined with 5-HT3 RA and dexamethasone can significantly reduce the incidence of full-cycle CINV, while the HXZQ oral solution group showed a greater effect in preventing delayed CINV. With the exception of a small number of subjects who reported a certain degree of discomfort in relation to the odor and taste of HXZQ, no unanticipated serious toxic side effects were observed during the trial, and the safety of HXZQ oral liquid was satisfactory. The reproducibility of the results was supported by the data from the phase IIB study of HXZQ oral liquid.^[15]^

Despite the commonality of the symptoms of nausea and vomiting, the specific physiological mechanisms that induce them and their associated risk factors remain poorly understood. It is unfortunate that a significant number of antiemetic medications currently available have limited effectiveness in relieving chemotherapy-induced nausea.^[20]^ Therefore, NNDs beyond the risk period was utilized as a study endpoint in this investigation. The mean NNDs was 13.23 in the group HX, which was significantly higher than that in group C (*P* = .004). In comparison with the group C, the HXZQ oral solution was found to provide a more significant alleviation of nausea symptoms.

The composition of Huo Xiang Zheng Qi Oral Liquid is as follows: Atractylodes macrocephala, Pericarpium Citri Reticulatae, Magnolia officinalis, Angelica Dahurica, Poria, Pinellia ternate, Pogostemon cablin, Licorice Infusion, Patchouli Oil, Perilla Leaf Oil. In the theoretical framework of Chinese medicine, Huo Xiang Zheng Qi Oral Liquid is believed to dispel exterior pathogens, resolve dampness, and regulate the flow of qi to harmonize the middle burner.^[21]^ The drug is primarily employed in the treatment of external wind-cold and internal dampness stagnation. Semixia and Chenpi in HXZQ have been shown to dry dampness and harmonize the stomach, thereby reducing rebelliousness and vomiting. Modern medical research has found that quercetin (GHX06), the active ingredient in HXZQ, is a typical flavonoid with significant antioxidant and anti-inflammatory effects, which can alleviate gastrointestinal inflammation. Indeed, GHX06 has been demonstrated to downregulate several pro-inflammatory mediators, such as TNF-α and IL-1β, in model rats, thereby exerting its anti-inflammatory function.^[22]^ The substances 5-HT3 and P, along with their corresponding receptors, have been identified as pivotal regulators of CINV. Concurrently, these substances have been demonstrated to function as mediators of gastrointestinal inflammation.^[23]^ Betulin (DFP01), an alkaloid component in HXZQ, has been demonstrated to promote intestinal peristalsis and enhance gastrointestinal motility. Furthermore, ion channels in smooth muscle cells, such as KCNH2 and SCN5A, are imperative for the regulation of gastrointestinal motility. Chuan Chenpianin (CP03), a constituent of HXZQ, interacts with these ion channels to regulate gastrointestinal motility by reflexing highly contractile gastrointestinal smooth muscles and exciting lowly contractile gastrointestinal smooth muscles. The utilization of Citrus sinensis peel, the raw material of chenpianin, has been observed to promote gastric emptying in murine models. In addition, the aqueous extract and volatile oil of Citrus sinensis peel have demonstrated the capacity to antagonize 5-HT, histamine, and acetylcholine-induced contraction of intestinal smooth muscle, thereby inhibiting the spontaneous movement of small intestinal smooth muscle and contributing to the regulation of vomiting.^[22,24]^ Lee et al.^[25]^ investigated the effects of acyclic 30-carbon precursor triterpenoids from Poria, a component of HXZQ, on the 5-HT3 receptor channel. The study found that these triterpenoids, in conjunction with 5-HT, reduced the inward current induced by the 5-HT3 receptor, thereby inhibiting the vomiting process.

Recent preclinical findings provide further biological plausibility for these antiemetic and gastrointestinal regulatory effects. In a Clostridioides difficile–induced colitis model, Huo Xiang Zheng Qi Liquid significantly improved survival, alleviated intestinal inflammation, and restored microbial homeostasis – showing efficacy comparable to vancomycin treatment.^[26]^ This suggests that beyond neurotransmitter modulation, HXZQ may also act through anti-inflammatory and microbiota-stabilizing pathways to protect the gastrointestinal mucosa. These multifaceted effects theoretically contribute to the reduction of chemotherapy-induced gastrointestinal reactions, aligning with its observed clinical benefits in CINV prevention.

In recent years, evidence has accumulated showing that many traditional medicine–derived compounds act through clearly defined biochemical and electrophysiological pathways, which provides a broader pharmacological context for HXZQ. For example, certain “toxic medicines” used in traditional oncology have been shown to regulate ion channels involved in tumor growth and neural signaling.^[27]^ Similarly, biomarker-driven studies have demonstrated that herbal compounds such as berberine, curcumin, and resveratrol can counteract histone deacetylase inhibitor–induced oncogenic signaling in esophageal squamous cell carcinoma.^[28]^ Beyond oncology, other TCM-derived agents like Icaritin and Epimedium extracts have been shown to activate ERK1/2–p38 and Notch pathways to promote osteoblastic differentiation and improve osteoporosis outcomes,^[29,30]^ while Hypericum perforatum exerts antidepressant effects by modulating AKT1, MAPK1, and immune-inflammatory pathways.^[31]^ Collectively, these findings illustrate how TCM interventions engage well-characterized molecular targets across organ systems.

The gastrointestinal and immune dimensions are particularly relevant to CINV. Studies have shown that Syzygium aromaticum can alleviate Helicobacter pylori–induced gastric inflammation by modulating TLR4/MyD88/NF-κB and MAPK pathways.^[32]^ Similarly, traditional medicines have been used to enhance organ recovery and immune modulation in posttransplant settings, highlighting their systemic applicability in clinical medicine.^[33]^ These cross-disciplinary findings support the biological plausibility of HXZQ’s antiemetic effect, reflecting a convergence between traditional gastrointestinal regulation and modern molecular immunology.

In recent years, an increasing number of herbal medicines have been used as complementary and alternative treatments for CINV. For instance, research conducted on astragalus polysaccharide has demonstrated its efficacy in enhancing the quality of life of patients afflicted with advanced non-small-cell lung cancer, while concurrently providing substantial relief from nausea and vomiting.^[34]^ A multicentre, double-blind, randomized controlled trial that utilized solely ginger powder^[35]^ as a treatment for CINV concluded that the efficacy and safety of the intervention was proven. In addition, Wu et al achieved good efficacy in the prevention of CINV in advanced colorectal cancer by using the Chinese herbal medicine, Hezhong granules.^[36]^ Rikkunshito, a Japanese herbal medicine, has been employed in the treatment of dyspepsia and anorexia. In a study conducted by Ohnishi et al., it was also used as a treatment for CINV in a cisplatin-combined paclitaxel chemotherapy regimen. This resulted in a complete remission rate that was significantly higher in the Rikkunshito group than in the control group. Rikkunshito has been demonstrated to antagonize 5-HT_2B_ and 5-HT_2C_ receptors in mice, producing effects similar to those of 5-HT antagonists. The effects observed in mice were similar to those of 5-HT antagonists, and this was due to the activity of the 5-HT_2B_ and 5-HT_2C_ receptors.^[37]^ Among other things, Rikkunshito contains Atractylodes macrocephala, Panax ginseng, Panax quinquefolium, sour dates, licorice and ginger. The primary components of the Hezhong granules utilized by Wu et al.^[36]^ comprised Banxia (Pinellia ternata), ginger, and Scutellaria baicalensis. Extracts of these components were found to be effective in inhibiting acute and delayed emesis in animal models, with this effect being achieved through the inhibition of central or peripheral NK1 receptors. Xiaohuansha Tang, a formulation comprising ginger and Banxia was demonstrated to be associated with the activation of the AMPK-Nrf2 signaling pathway and the restoration of cisplatin-induced PINK1/Parkin-mediated mitogenic defects in the gastrointestinal tract.^[38]^ The HXZQ used in this study also contained Banxia (Pinellia ternata), and the authors hypothesized that its mechanism in alleviating CINV might be similar.

It is noteworthy that the assumption of a 4-day improvement in mean NCDs used for sample size estimation was derived from our previous phase II clinical study, in which Huo Xiang Zheng Qi Oral Liquid combined with standard antiemetic therapy increased the average NCDs by approximately 4 days compared with the control regimen. Therefore, this value was adopted as the expected effect size for the present phase III trial. In retrospect, the target may have been somewhat optimistic; however, it provided a scientifically grounded and clinically meaningful benchmark. Even small gains in NCDs can translate into tangible benefits for patients – fewer days of discomfort, better appetite and sleep, and greater treatment adherence – highlighting that incremental progress in CINV prevention can substantially improve quality of life during chemotherapy.

Notwithstanding the favorable efficacy of this study in alleviating CINV, there are still some nonnegligible limitations. Firstly, the sample size was limited, with 27 (16.3%) subjects withdrawing from the study for various reasons, resulting in a final inclusion total of 139 subjects, which may restrict the generalisability of the results. A larger sample size is required to further validate the efficacy of HXZQ in subsequent follow-up studies. Secondly, only 1 chemotherapy cycle was assessed, living the efficacy of HXZQ in subsequent cycles unclear. The role of prevention and treatment is not clear, especially for refractory CINV. Thirdly, NK1RAs was not included in the health insurance at the time of the study’s design and launching phase, resulting in the continuation of the intervention from the previous phase IIB study.^[15]^ Therefore, dexamethasone + 5HT3RAs + HXZQ/placebo prophylaxis was utilized. However, NK1RAs are now integrated into the national health insurance scheme, resulting in a significant improvement in clinical accessibility. The findings of this study do not provide sufficient evidence to support the HXZQ combined with NK1RAs regimen. Further research in this area is therefore highly recommended. In addition, minor differences in taste and odor between HXZQ and placebo may have led to partial unblinding, thus representing a potential source of performance bias.

In subsequent studies, the objective is to augment the sample size to encompass a more extensive cohort of patients with diverse treatment histories, with the aim of investigating the long-term efficacy of the intervention in patients undergoing multiple cycles of chemotherapy. The present study will be followed by in-depth mechanistic investigations into the role of HXZQ in the remission of CINV. Furthermore, a forthcoming clinical trial will explore whether HXZQ, when combined with NK1RAs, dexamethasone, and 5-HT3RAs, can effectively prevent CINV during multiday HEC regimens.

## 6. Conclusion

In patients without access to NK1RAs, HXZQ in combination with a 5-HT3 receptor antagonist and dexamethasone is a safe and feasible regimen compared to placebo for better prevention of CINV due to 3-day cisplatin-containing HEC chemotherapy throughout the entire chemotherapy cycle.

## Acknowledgments

We are profoundly grateful to the 166 patients who entrusted us with their care during this trial. Our sincere appreciation extends to the clinical staff across all 10 participating oncology centers, whose collective effort made this multicenter collaboration possible. We particularly acknowledge the contributions of Chengdu Fifth People’s Hospital, Jiangyou 903 Hospital and all collaborating sites in Southwest China for their dedication to high-quality data collection and protocol adherence. This study represents the shared endeavor of ten oncology units, and its completion would not have been possible without the joint dedication and collaboration of our core team. We extend our deepest gratitude to Songze Wu, Zixuan Ye, Ying Wang, and Juan Chen, whose sustained contributions were vital in study conception, data acquisition, and manuscript preparation. We also sincerely acknowledge Jun He, Lang He, and Yu Sun whose equal leadership, guidance, and coordination played a decisive role in shaping the study design, analysis, and interpretation. Although authorship designation does not formally reflect shared senior or corresponding responsibility, we wish to express our special appreciation for their equally essential intellectual and supervisory contributions to this work. We also acknowledge Taiji Group Chongqing Fuling Pharmaceutical Co. for providing Huo Xiang Zheng Qi Oral Liquid compliant with the 2020 Pharmacopoeia of the People’s Republic of China standards (Approval No. Z50020409, State Food and Drug Administration). The company had no role in study design, data collection, or analysis.

## Author contributions

**Conceptualization:** Jiang Zhu, Jun He.

**Data curation:** Liping Tong, Ying Wang, Juan Chen, Ting Li, Na Li, Taifang Peng, Liqin Xia, Lang He.

**Formal analysis:** Jiang Zhu, Songze Wu, Yangang Zhou, Jie Xian, Jun He, Lang He.

**Investigation:** Jie Xian, Lang He.

**Methodology:** Yu Sun.

**Project administration:** Jiang Zhu, Songze Wu, Zengjin Hu.

**Resources:** Taifang Peng.

**Supervision:** Xianguo Liu, Zhiying Yue, Jun He, Yu Sun.

**Visualization:** Na Li.

**Writing – original draft:** Zixuan Ye.

**Writing – review & editing:** Yu Sun.
